# Proinflammatory Cytokines and C-Reactive Protein in Uveitis Associated with Behçet's Disease

**DOI:** 10.1155/2014/396204

**Published:** 2014-06-08

**Authors:** Marina Mesquida, Blanca Molins, Victor Llorenç, Maite Sainz de la Maza, María Victoria Hernandez, Gerard Espinosa, Alfredo Adán

**Affiliations:** ^1^Institut Clínic d'Oftalmologia, Hospital Clinic de Barcelona, University of Barcelona, Sabino de Arana 1, 08028 Barcelona, Spain; ^2^Institut d'Investigacions Biomèdiques August Pi i Sunyer (IDIBAPS), Barcelona, Spain; ^3^Rheumatology Department, Hospital Clinic de Barcelona, University of Barcelona, Barcelona, Spain; ^4^Autoimmune Disease Department, Hospital Clinic de Barcelona, University of Barcelona, Barcelona, Spain

## Abstract

The aim of the present study was to determine the serum cytokine profile and levels of high sensitivity C-reactive protein (hsCRP) in patients with uveitis associated with Behçet's disease (BD) and to compare them with those obtained from healthy control subjects. We determined the serum concentration of interferon-*γ* (IFN-*γ*), interleukin-1*β* (IL-1*β*), IL-12p70, IL-17A, tumor necrosis factor-*α* (TNF-*α*), and hsCRP in 13 patients with active uveitis associated to BD, 24 inactive BD patients, and 20 controls. In a subgroup of 10 active patients, a second serum sample was obtained when the disease was inactive. Cytokine profiles and hsCRP levels were correlated with disease activity, severity, complications, and visual outcome. Levels of IFN-*γ* and TNF-*α* were significantly increased in patients with active uveitis associated to BD compared to controls (*P* < 0.05). IFN-*γ*, TNF-*α*, and hsCRP were significantly higher during active uveitis associated to BD compared to inactive disease (*P* < 0.05). Furthermore, IL-17A was significantly increased in patients with active BD without pharmacological treatment compared to controls (*P* < 0.05). No significant correlations were found with specific cytokine profiles and disease severity, visual outcome, or complications. In summary, increased serum levels of IFN-*γ*, TNF-*α*, IL-17A, and hsCRP were associated with active uveitis associated with BD and might serve as markers of disease activity.

## 1. Introduction

Behçet's disease (BD) is a chronic systemic inflammatory disorder at the crossroad between autoimmune and autoinflammatory syndromes. Major symptoms include oral aphthous ulcers, genital ulcerations, skin lesions, and ocular lesions [1]. Uveitis, associated with BD, is a sight-threatening condition that affects 60–80% of BD patients and is characterized in its more severe form by posterior or panuveitis including occlusive retinal vasculitis [2]. Although the pathogenesis of BD remains poorly characterized, it is currently believed that certain infectious and/or environmental factors are able to trigger symptomatology in predisposed individuals [2]. Association with Class I MHC (HLA-B*51) may predispose to inflammation with engagement of the innate immune system, and further perpetuation by the adaptive T cell responses against infectious antigens and/or autoantigens [1, 3]. The aim of therapeutic strategy in BD is to prevent the recurrent inflammatory attacks in order to minimize potential irreversible damage. Systemic corticosteroids (CS) and immunosuppressive (IS) drugs are the first line of treatment for severe manifestations of BD [4, 5] but often do not result in stable remission and a subgroup of patients keep on suffering flare-ups despite conventional therapy [6]. In these cases, novel biologic response modifier (BRM) drugs, particularly TNF-*α* antagonists, have emerged as a valid option resulting in substantial improvement of long-term visual prognosis and patients' quality of life [7].

The current hypothesis for the pathogenesis of BD states that genetic factors induce a general hyperactivity of the immune system and bacterial or viral infection stimulates the expression of heat shock proteins (HSP) and MICA (MHC Class I chain-related molecules A). This induces the upregulation of adhesion molecules in the endothelium (ICAM-1 and VCAM-1), activation of coagulation, and stimulation of T cells by secreting IL-6, IL-8, and MCP-1 among others, continuing elevation of the cytokine production eventually leading to tissue damage and vasculitis (reviewed in [8]). Previous studies suggested that BD pathogenesis is predominated by a T helper (Th)1 and Th17 immune response [9–13] as increased levels of IFN-*γ*, IL-12, and TNF-*α* have been found in patients with BD [10]. Active BD was also shown to be characterized by increased levels of IL-17A [11–13]. Furthermore, genetic studies including genome-wide association studies, identified IL23R-IL12RB2 and IL10 as BD susceptibility loci [14, 15]. Therefore, Th1/Th17-type immune responses may play a critical role in BD. However, it is unclear whether Th1/Th17-related cytokines could serve as biomarkers of disease activity and the effect of both standard and novel therapies on the circulating levels of these cytokines in BD patients is unknown. Moreover, it is also ill-defined whether uveitis associated with BD is characterized by altered levels of acute phase reactants such as high-sensitivity (hs) C-reactive protein (CRP) that may eventually exacerbate the inflammatory burst further modifying serum inflammatory cytokine levels.

In the present study, we used a multiplex assay to study the profile of 5 cytokines and hsCRP in serum samples obtained from patients with active and inactive BD-associated uveitis and an age-matched healthy control group. In a subset of patients, the cytokine profile was analyzed longitudinally during active and inactive stages of the disease. Additionally, cytokine profiles were correlated with visual outcome, disease severity, and ocular complications.

## 2. Materials and Methods

### 2.1. Subjects

Patients were recruited from the Ophthalmology Department, Hospital Clinic of Barcelona (Spain), between January 2011 and July 2013. Thirty-seven patients aged 22–71 years (mean age: 40.6 ± 10.8) with uveitis associated with BD were invited to participate in the study. Twenty-two patients were male and 15 were female. Thirty-two patients were Caucasians and 5 were from North of Africa.

Inclusion criteria were patients diagnosed with BD fulfilling the diagnostic criteria of the International Study Group [16] who presented with intraocular inflammation. The diagnosis of active disease followed the clinical criteria based on inflammatory cell reaction in the anterior chamber or vitreous > 0.5+ as per Standardization of Uveitis Nomenclature (SUN) and National Eye Institute (NEI) grading system [17, 18]. Active retinal lesions and retinal vasculitis were evaluated by indirect ophthalmoscopy, fundus autofluorescence, and/or fundus fluorescein angiography. Any mentioned inflammatory sign (i.e., anterior chamber cell > 0.5+, vitreous cells > 0.5+, active retinal vasculitis, or active chorioretinal lesions) was enough to be eligible. Ocular complications included macular edema; tractional, serous, or rhegmatogenous retinal detachment; retinal tear; retinal neovascularization; epiretinal membrane; persistent vitreous opacities; vitreous hemorrhage; and optic atrophy. BD was considered severe when it included any of the mentioned complications.

Visual acuity was measured with Snellen charts and was converted to logarithm of the minimum angle of resolution (log-MAR) values for statistical analysis.  Table 1 summarizes different features of patients and controls. Immunomodulatory treatments that patients were receiving (i.e., CS, IS, and/or BRM) were also gathered.

For comparison, 20 age-matched (mean age: 40.1 years) and gender-matched (10 women and 10 men) healthy subjects (by clinical anamnesis, ocular examination, and complete blood analysis), with no evidence of active ocular disease, who were waiting for unrelated ophthalmic surgery (cataract, eyelid benign lesions, etc.), were recruited as a control group.

All patients and controls provided informed consent and the research followed the tenets of the Declaration of Helsinki. The Hospital Clinic of Barcelona Institutional Review Board (IRB) approved this study according to local and national IRB guidelines.

Blood was collected aseptically just after ophthalmological evaluation and serum was prepared and stored at −70°C until further cytokine analysis. A total of 13 samples were obtained from patients with active uveitis and 24 samples were collected from patients with inactive disease. In a subset of 10 patients, the cytokine profile was analyzed longitudinally during active and inactive stages of the disease during a mean follow-up of 7.2 ± 1.1 months.

### 2.2. Serum Cytokine Determination

The Luminex platform (Millipore's MILLIPLEX Human Cytokine/Chemokine kit) was used to measure serum cytokine levels as recommended by the manufacturer. Five immune mediators associated with Th1 and Th17 responses (IFN-*γ*, IL-12p70, IL-17A, IL-1*β*, and TNF-*α*) were determined. These cytokines were chosen based on the results of a preliminary study (data not shown) where 10 cytokines were analyzed (IFN-*γ*, IL-1*β*, IL-2, IL-4, IL-6, IL-8, IL-10, IL-12(p70), IL-17A, and TNF-*α*). The cytokine and chemokine assay plate layout consisted of 7 standards in duplicate (3.2–2,000 pg/mL), 1 blank well (for background fluorescence subtraction), 2 internal quality control samples in duplicate, and 25 *μ*L duplicates of each serum sample.

### 2.3. Determination of Levels of C-Reactive Protein

Levels of hsCRP in serum samples were determined by CRP high sensitivity ELISA (hsCRP, IBL International GMBH) following the manufacturer's instructions.

### 2.4. Statistical Analysis

Nonparametric analysis was performed using the Mann-Whitney test for comparison of unpaired data from two groups and Wilcoxon test for comparison of paired data. Differences among the three groups were evaluated using the Kruskal-Wallis test. Spearman's correlation analysis was carried out to determine the association of serum cytokine levels with clinical parameters. Statistical significance was set at *P* < 0.05. All calculations were performed using SPSS Version 18.0 (SPSS, IBM Corporation, New York).

## 3. Results

Thirty-seven patients (22 male and 15 female) with uveitis associated with BD were included in the study. When serum sample was obtained, patients with active uveitis associated with BD were receiving the following therapies: 3/13 patients were not receiving any immunomodulatory treatment (naïve subjects with first episode of uveitis attack), 7/13 were treated with BRM (TNF-*α* antagonists), 2/13 were receiving CS, and 1/13 was receiving conventional IS therapy. With regard to the inactive patients group, 13/24 were not receiving any immunomodulatory treatment due to long-standing remission, whereas 2 patients were under low-dose CS, 8 patients were receiving BRM (TNF-*α* antagonists), and one patient was on IS therapy.

**Figure 1:**
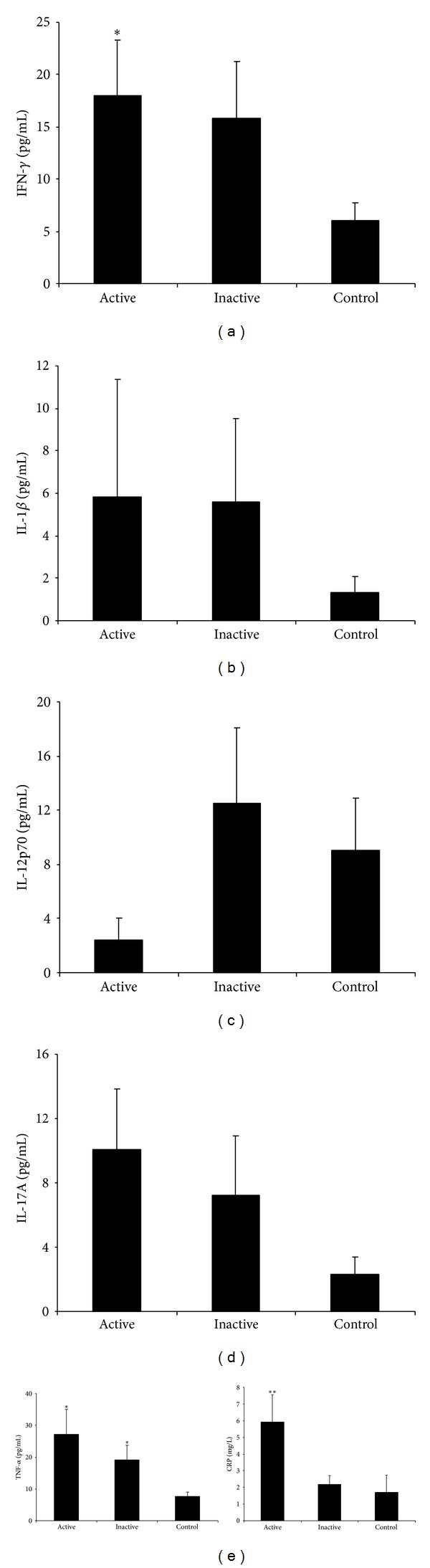
Cytokine profile and hsCRP levels of serum samples of patients with active BD (*N* = 13), inactive ocular BD (*N* = 24), and control subjects (*N* = 20). Results are expressed as mean values of cytokine levels (pg/mL) ± SEM. Statistical analysis was performed by Kruskal-Wallis test (*P < 0.05 versus control; **P < 0.05 versus control and inactive).

Firstly, we analyzed differences in the serum cytokine profile among patients with active disease, inactive disease, and healthy subjects. For this purpose the cytokine levels of 13 patients with active ocular BD, 24 different patients with inactive BD, and 20 controls were determined.  Figure 1 shows IFN-***γ***, IL-12p70, IL-17A, IL-1*β*, and TNF-*α* serum cytokine levels from each group. Active patients showed the highest levels of IFN-**γ**, IL-17A, IL-1*β*, and TNF-*α*, whereas the control group had the lowest levels of these cytokines. IFN-**γ** and TNF-*α* levels were significantly higher in active patients compared to control subjects (Figures 1(a) and 1(e); *P* < 0.05). In fact, IFN-**γ** correlated well with IL-17A (Spearman's rho *r* = 0.833; *P* < 0.01) and with TNF-*α* (*r* = 0.855; *P* < 0.01) in active patients. TNF-*α* also correlated well with IL-17A (*r* = 0.663; *P* < 0.05). Patients with active disease also showed significantly higher levels of hsCRP compared to inactive patients (5.91 ± 1.65 mg/L active versus 2.19 ± 0.53 mg/L inactive; *P* = 0.029).

**Figure 2:**
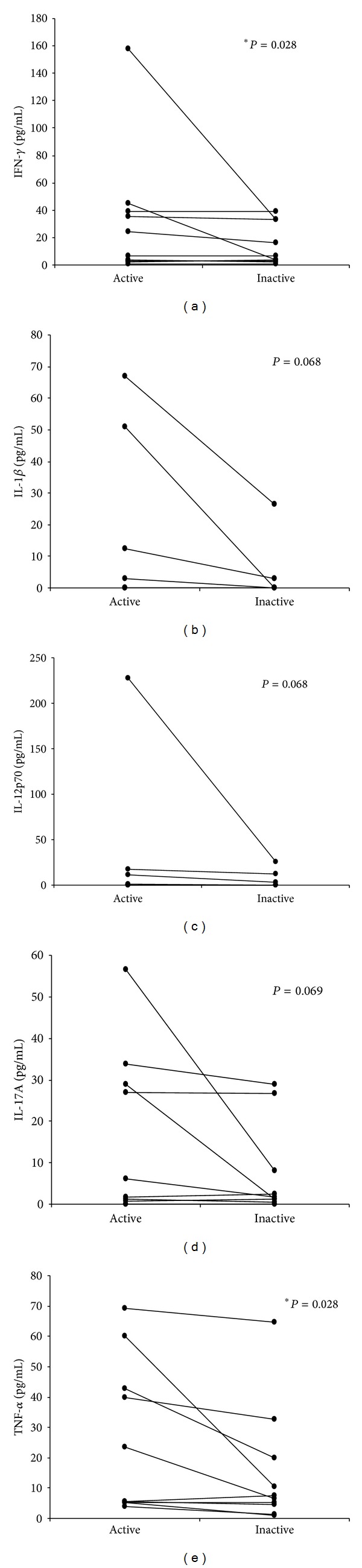
Levels of IFN-*γ*, IL-12p70, IL-17A, IL-1*β*, and TNF-*α* (pg/mL) ± SEM in the serum of patients with uveitis associated with BD during the active and inactive stages (*N* = 10). Statistical analysis was conducted using the Wilcoxon test (*P < 0.05).

In a subgroup of 10 patients, serum samples were obtained during both active and inactive phases of the disease and, as shown in Figure 2, IFN-**γ** and TNF-*α* levels were significantly higher during the active phase compared to the inactive period.

**Figure 3:**
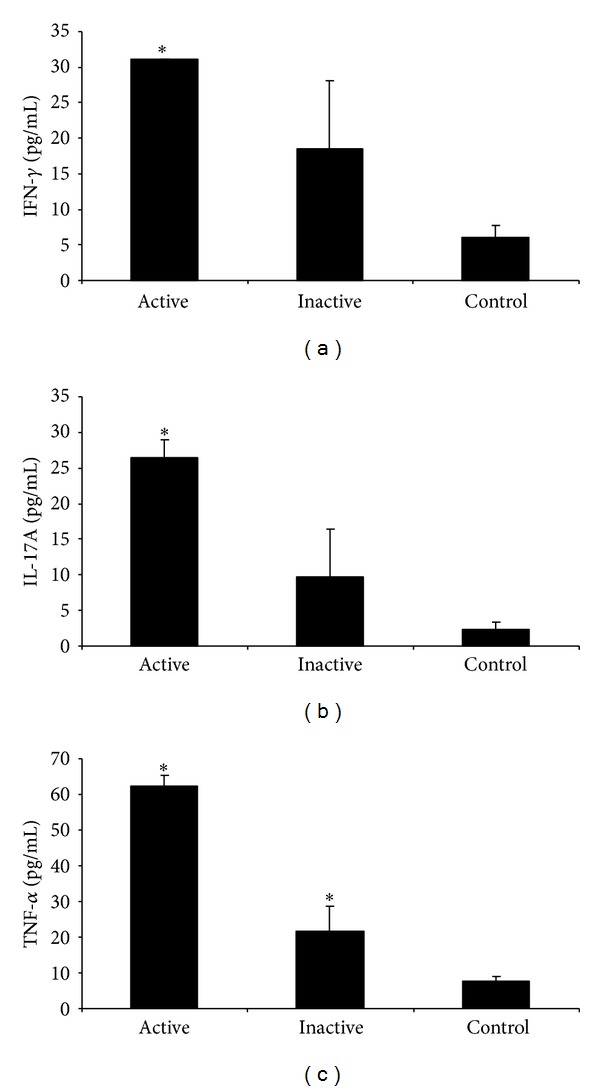
Circulating levels of IFN-*γ*, IL-17A, and TNF-*α* in patients with active BD (*N* = 3) and inactive BD (*N* = 13) without treatment and control subjects (*N* = 20). Results are expressed as mean values of cytokine levels (pg/mL) ± SEM. Statistical analysis was performed by Kruskal-Wallis test (*P < 0.05).

Interestingly, as shown in Figure 3, when considering only those samples from patients without any pharmacological treatment (*n* = 3 active and *n* = 13 inactive), IL-17A levels were also significantly higher in active patients compared to healthy subjects (Figure 3(b), *P* < 0.05). In addition, untreated inactive patients also showed significantly higher levels of TNF-*α* compared to healthy subjects (Figure 3(c), *P* < 0.05).

**Figure 4:**
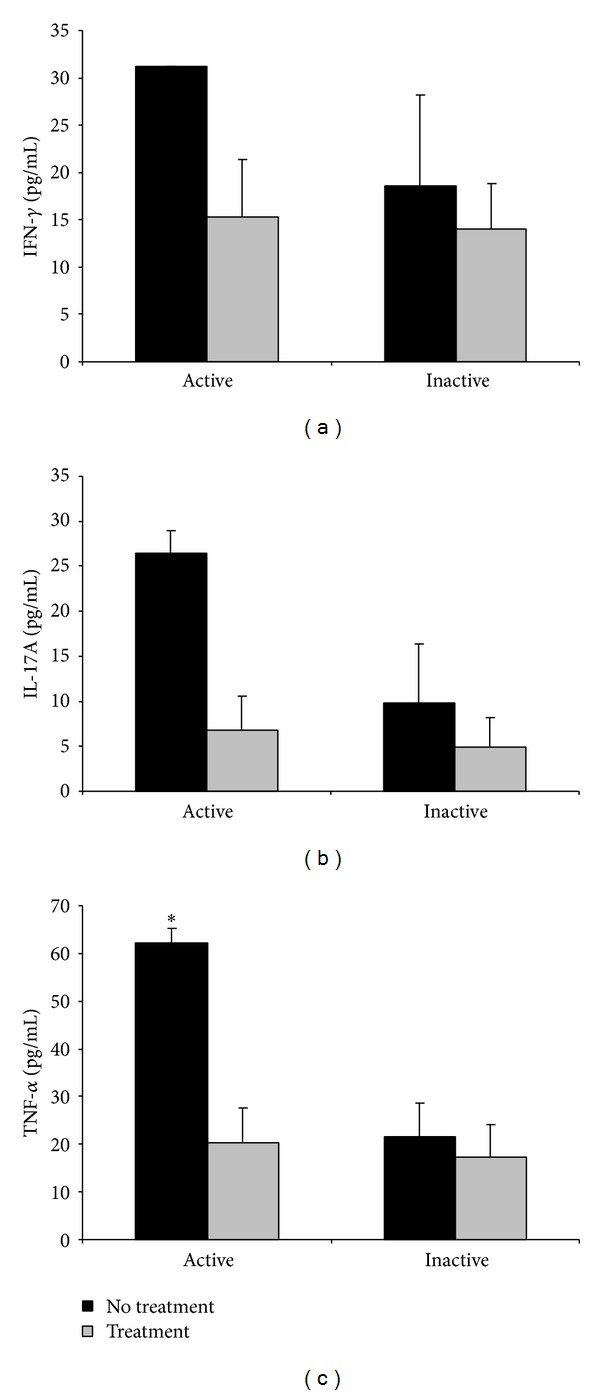
Effect of treatment on circulating levels of IFN-*γ*, IL-17A, and TNF-*α* (pg/mL) ± SEM in patients with active and inactive BD. Results are expressed as mean values of cytokine levels (pg/mL) ± SEM. Statistical analysis was performed by Mann Whitney* U*-test (*P < 0.05).

We then analyzed the effect of pharmacological treatment on the circulating levels of the different cytokines. Thus, serum cytokine levels in active and inactive patients were compared between patients with or without treatment. As observed in Figure 4, IFN-*γ*, IL-17A, and TNF-*α* levels were higher in patients without treatment, although only TNF-*α* levels in active patients with no treatment were statistically different from those of active patients with treatment (Figure 4(c)). However, we were unable to observe differences in any of the cytokines among the different treatments (data not shown).

Finally, none of the analyzed cytokines correlated with visual acuity or disease severity in neither active nor inactive BD patients and there were no significant differences between cytokine or hsCRP levels of patients with occlusive vasculitis (*n* = 5) and those without complications (data not shown).

## 4. Discussion

Behçet's disease (BD) is a chronic multisystemic inflammatory disease, where autoimmunity seems to play a crucial role [19, 20]. Ocular involvement affects around 70% of BD patients [21] and is characterized by relapsing nongranulomatous uveitis involving both the anterior and the posterior segments of the eye. These ocular lesions are often sight-threatening, requiring prompt and aggressive treatment to preserve vision [22]. Although several reports have suggested involvement of Th1 and Th17 immune responses in BD [9, 10], it is still unclear whether Th1- and Th17-related cytokines may serve as markers of disease activity, severity, and/or visual outcome. In the present study we aimed to study the serum cytokine profile and hsCRP levels in patients with uveitis associated with BD and its relationship with visual outcome and disease severity. We showed that IFN-*γ*, IL-17A, TNF-*α*, and hsCRP are increased in active uveitis associated with BD.

We observed that BD patients with active uveitis showed significantly higher levels of IFN-*γ* and TNF-*α* compared to healthy subjects. These results are in agreement with previously published observations that reported increased levels of IFN-*γ* and TNF-*α* in active BD without treatment, thus supporting a Th1 immune response [9, 10]. Interestingly, we observed these effects not only in patients without any treatment but also in those undergoing pharmacological treatment. Moreover, the analysis of the cytokine profile in the subgroup of patients to whom we obtained serum during both active and inactive stages also revealed that IFN-*γ* and TNF-*α* significantly increased during the active stage of the disease. Similarly, IL-17A was increased in patients with active uveitis compared to healthy controls. However, this increase was statistically significant only in those patients without treatment. These observations are in agreement with other groups who found increased levels of circulating IL-17A in patients with active BD compared to healthy subjects [11, 12], supporting the involvement of Th17 response in BD. We observed that IFN-*γ* correlated well with TNF-*α* and IL-17A in patients with active BD. Additionally, TNF-*α* also correlated well with IL-17A in these patients. These observations support the idea that BD is mediated simultaneously by both Th1 and Th17 responses. On the other hand, although IL-1*β* and IL-12 seemed to be increased in active BD, the difference did not reach statistical significance and none of the analyzed mediators correlated with visual outcome or disease severity. Similarly, we were unable to detect differences in the cytokine profile of BD patients with occlusive vasculitis and in those without.

In the present work we also observed significantly higher levels of circulating hsCRP in patients with active uveitis associated with BD compared to inactive patients. CRP is the prototypical acute phase reactant and an active regulator of the innate immune system. It is considered to be a serum biomarker for chronic inflammation [23]. Indeed, CRP was significantly higher in active patients compared not only to healthy subjects but also to inactive patients. Among the many functions ascribed to CRP are activation of the classical complement pathway and inactivation of the alternative pathway [24]. In plasma, CRP exists as a cyclic pentamer. However, CRP can undergo dissociation, upon exposure to acidic or inflammatory conditions, thereby acquiring distinct functionality [25, 26]. In fact, dissociated monomeric CRP has been shown to display a proinflammatory phenotype [27–30]. Therefore, the increased levels of circulating hsCRP in patients with active uveitis associated with BD could eventually contribute to the exacerbation of the inflammatory burst increasing the secretion of Th1- and Th17-proinflammatory cytokines.

The aim of therapeutic strategy in BD is to prevent recurrent inflammatory attacks in order to minimize potential irreversible damage. As mentioned above, classical treatment with systemic CS and conventional IS therapies may be effective in some cases [5]. Nonetheless, there is a subgroup of patients who keep on suffering flare-ups despite standard therapy. Thus, in the present work, we also aimed to analyze the effect of pharmacological treatment on the cytokine profile of BD. Indeed, we observed that IFN-*γ*, TNF-*α*, and IL-17A levels were higher in patients without treatment than in those with pharmacological treatment in both active and inactive patients. Nevertheless, we did not observe differences in the cytokine profile among the different treatments. Our observations suggest that successful therapies should be able to prevent recurrent inflammatory attacks by keeping under check the circulating levels of these proinflammatory cytokines. However, inactive patients maintained significantly higher levels of TNF-*α* than controls, thus keeping a proinflammatory background.

Our cohort of patients with uveitis associated with BD was very representative as it included patients with different demographic origins. In fact, we did not observe differences in the cytokine profile between Caucasian and African patients (data not shown), which allows us to speculate that the observed increased levels of IFN-*γ*, TNF-*α*, and IL-17A may apply to uveitis associated with BD from any origin. The main limitation of the present work is the sample size, specially the group of active patients without treatment as well as the heterogeneity of treatment modalities. Nevertheless, the fact that our results were observed across such a heterogeneous group also reinforces our findings.

## 5. Conclusions

In conclusion, our study shows that active BD is associated with increased serum levels of IFN-*γ*, TNF-*α*, IL-17A, and hsCRP compared to inactive disease or healthy controls. Although further research is warranted to elucidate the role of these mediators in BD, serum cytokine profiling may contribute to the understanding of the physiopathology processes underlying retinal damage in BD and provide tools for new biomarkers and/or personalized treatment targets.

## Figures and Tables

**Table 1:** Clinical characteristics of BD patients and normal controls.

Parameters	Normal controls (*n* = 20)	Active BD patients (*n* = 13)	Inactive BD patients (*n* = 24)
Age (years)	40,1 ± 4,7	31,7 ± 6,8	44,9 ± 9,7
Female/male (*n*)	10/10	6/7	9/15
Anterior uveitis	NA	0/13	4/24
Posterior uveitis	NA	5/13	12/24
Panuveitis	NA	8/13	8/24
Cells in anterior chamber	NA	8/13	0/24
Vitreous cells	NA	13/13	2/24
Retinal vasculitis	NA	10/13	0/24
Oral ulcers*	NA	11/13	2/24
Genital organ ulcers*	NA	9/13	1/24
Erythema nodosum*	NA	4/13	0/24
Arthritis*	NA	2/13	0/24
Visual acuity (logMAR)	ND	0,36 ± 0,30	0,45 ± 0,73

Data are shown as mean ± SD or absolute numbers.

*Extraocular lesions observed at the time of blood sampling are shown in this table. Patients whose extraocular lesions were negative may have experienced these symptoms during an earlier phase of the disease.

BD: Behçet's disease; NA: not applicable; ND: not determined.
